# Corrigendum to “Comparing Types of Yoga for Chronic Low Back and Neck Pain in Military Personnel: A Feasibility Randomized Controlled Trial”

**DOI:** 10.1177/27536130231161197

**Published:** 2023-03-15

Groessl EJ, Casteel D, McKinnon S, et al. Comparing Types of Yoga for Chronic Low Back and Neck Pain in Military Personnel: A Feasibility Randomized Controlled Trial. *Global Advances in Health and Medicine*. 2022;11. doi:10.1177/2164957X221094596

In the above-mentioned article, the author name should read as “Douglas G Chang”.

The correction has been updated in the online version.

